# Omental Torsion: A Rare Mimicker of Acute Appendicitis

**DOI:** 10.7759/cureus.76966

**Published:** 2025-01-05

**Authors:** Oscar Antonio Regalado Morales, Paul Montoya Alarcón, Luis Alberto Solís García, José Luis Herrera Alanís, Liliana Bello Saucedo

**Affiliations:** 1 Radiology, ISSSTE Regional Hospital Monterrey, Monterrey, MEX

**Keywords:** abdominal computed tomography, abdominal radiology, acute abdomen, acute pancreatitis (ap), omental torsion

## Abstract

Omental torsion is one of the less common causes of abdominal pain, with symptoms that may be indistinguishable from more frequent pathologies, such as acute appendicitis. Computed tomography (CT) is the diagnostic modality of choice due to its characteristic findings for this condition. We report the case of a 51-year-old female with omental torsion presenting with clinical and physical examination findings indistinguishable from acute appendicitis.

## Introduction

Acute abdomen is one of the most common reasons for emergency hospital care, accounting for up to 11% of cases [1]. Among the causes of this entity, omental torsion is one of the rarest, representing less than 1% of abdominal pain cases. Although it is a benign condition, its clinical diagnosis is challenging due to the similarity of its symptoms with other pathologies. CT is the imaging modality of choice for both confirming the diagnosis and excluding differential diagnoses [2].

In this report, we present the case of a 51-year-old female with omental torsion, whose clinical presentation mimicked acute appendicitis.

## Case presentation

A 51-year-old female presented with abdominal pain localized to the right lower quadrant, with 24 hours of evolution, initiated after a sudden movement on public transportation. On physical examination, there was tenderness on palpation and signs of peritoneal irritation. Given the clinical presentation, the initial diagnosis was acute appendicitis.

**Figure 1:**
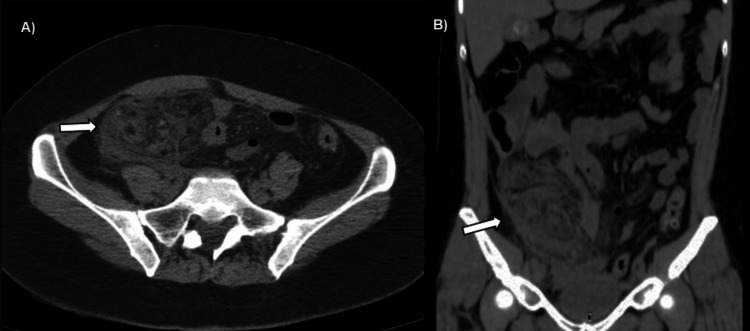
Computerized tomography in a simple phase of the abdomen. Axial (A) and coronal (B) CT scans show a rounded, partially defined, and heterogeneous mass with fat density localized to the right lower quadrant, adjacent to the anterior abdominal wall (thick arrow).

**Figure 2:**
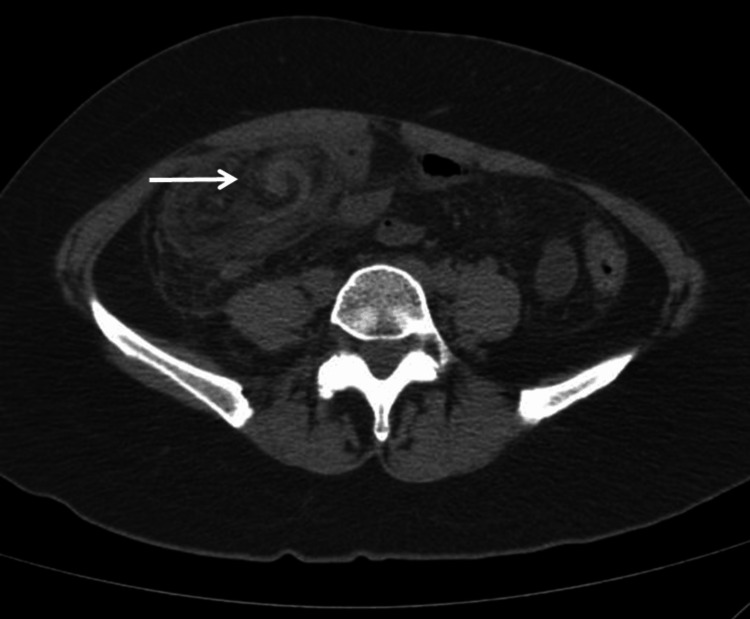
Computerized tomography in a simple phase of the abdomen. An axial CT scan shows elongated structures (blood vessels) within the mass forming a concentric circle pattern, characteristic of the whirl sign (thin arrow).

A non-contrast abdominal CT scan revealed a heterogeneous mass with fat density localized to the right lower quadrant, anterior to the intestinal loops and the cecum, extending to the pelvic cavity (Figures 1A, 1B). Additionally, multiple hyperdense linear images within the mass formed a concentric circle pattern, characteristic of the whirl sign (Figure 2). 

Detailed information about the treatments administered and the patient's follow-up is not available, as she chose to receive treatment at another institution, and it was not possible to access subsequent records. Despite this, the findings previously described in the case are significant, as they indicate omental torsion.

## Discussion

The greater omentum is an anatomical structure consisting of a four-layered peritoneal fold primarily composed of adipose tissue and small gastroepiploic vessels. It extends like an apron from the greater curvature of the stomach, covering the intestinal loops, and can move within the abdominal cavity [3].

Omental torsion is a condition characterized by the rotation of the greater omentum around its own axis. It can be classified as primary, when it occurs without predisposing factors, or secondary, when associated with underlying conditions [4]. Although an evident cause is not always identified, factors such as anatomical variants, obesity, sudden movements, and increased intra-abdominal pressure may increase the risk of its occurrence [5].

This condition typically affects middle-aged women, who often present with sudden-onset abdominal pain localized to the right lower quadrant, due to the greater size and mobility of the omentum in this region. Associated symptoms may include nausea, vomiting, and fever [6]. The most common laboratory findings are leukocytosis and elevated C-reactive protein (CRP) levels [1].

Imaging studies are crucial for diagnosis, given the wide spectrum of differential diagnoses. CT is the diagnostic modality of choice, as its findings are characteristic and help rule out other causes of acute abdomen. The main CT findings include a poorly defined heterogeneous mass with fat density, sometimes accompanied by free fluid. The whirl sign, considered pathognomonic, consists of hyperdense linear images within the mass, representing twisted blood vessels [7]. Other imaging modalities, such as ultrasound, may be useful in identifying a hyperechoic ovoid mass adherent to the anterior abdominal wall; however, their primary role is to exclude more common causes of abdominal pain [8].

The treatment of primary omental torsion includes two main approaches. The first is conservative management with analgesic therapy and antibiotic prophylaxis. If this fails, surgical treatment is indicated, typically via laparoscopy, which allows definitive diagnosis and resection of the affected portion of the omentum [2].

## Conclusions

Omental torsion is a rare and challenging diagnosis as its clinical presentation mimics more common conditions, such as acute appendicitis, diverticulitis, or epiploic appendagitis. Imaging studies are essential for accurate diagnosis, with CT being the modality of choice. Characteristic findings include a fatty mass and the whirl sign, which are considered classic features of this disease.
